# Biomimetic Artificial Muscles Inspired by Nature’s Volume-Change Actuation Mechanisms

**DOI:** 10.3390/biomimetics10120816

**Published:** 2025-12-04

**Authors:** Hyunsoo Kim, Minwoo Kim, Yonghun Noh, Yongwoo Jang

**Affiliations:** 1 DRB Research, DRB Industrial Co., Ltd., 28, Gongdandong-ro 55beon-gil, Busan 46329, Republic of Korea; kim.hyun.soo@drbworld.com; 2 Department of Medical and Digital Engineering, College of Engineering, Hanyang University, Seoul 04736, Republic of Korea; kmw5478@hanyang.ac.kr (M.K.); rlrr2121@hanyang.ac.kr (Y.N.); 3 Department of Pharmacology, College of Medicine, Hanyang University, Seoul 04736, Republic of Korea

**Keywords:** artificial muscle, biomimetic actuation, stimulus-responsive materials, volume-change mechanism, soft robotics

## Abstract

Artificial muscles translate the biological principles of motion into soft, adaptive, and multifunctional actuation. This review accordingly highlights research into natural actuation strategies, such as skeletal muscles, muscular hydrostats, spider silk, and plant turgor systems, to reveal the principles underlying energy conversion and deformation control. Building on these insights, polymer-based artificial muscles based on these principles, including pneumatic muscles, dielectric elastomers, and ionic electroactive systems, are described and their capabilities for efficient contraction, bending, and twisting with tunable stiffness and responsiveness are summarized. Furthermore, the abilities of carbon nanotube composites and twisted yarns to amplify nanoscale dimensional changes through hierarchical helical architectures and achieve power and work densities comparable to those of natural muscle are discussed. Finally, the integration of these actuators into soft robotic systems is explored through biomimetic locomotion and manipulation systems ranging from jellyfish-inspired swimmers to octopus-like grippers, gecko-adhesive manipulators, and beetle-inspired flapping wings. Despite rapid progress in the development of artificial muscles, challenges remain in achieving long-term durability, energy efficiency, integrated sensing, and closed-loop control. Therefore, future research should focus on developing intelligent muscular systems that combine actuation, perception, and self-healing to advance progress toward realizing autonomous, lifelike machines that embody the organizational principles of living systems.

## 1. Introduction

Nature has served as a fundamental source of inspiration for scientific and technological advancement for centuries. The field of biomimetics formalizes this concept by systematically translating the principles and mechanisms underlying biological systems into engineered designs. Among the most sophisticated natural systems, the muscle represents a paradigm of dynamic actuation capable of converting chemical energy into precisely controlled mechanical motion with exceptional efficiency and adaptability while also possessing an intrinsic self-healing capacity. The exceptional performance of biological muscles has inspired the development of biomimetic artificial muscles, which is a rapidly evolving research field focused on creating synthetic actuators that replicate the contractile behavior of their natural counterparts. These bioinspired systems have driven transformative advances across diverse disciplines, including soft robotics, adaptive and responsive materials, and biomedical engineering [1,2]. Recent advances in functional materials, particularly electroactive polymers (EAPs), carbon nanotubes (CNTs), and other stimuli-responsive nanostructures, have significantly accelerated the development of artificial muscles capable of bridging the gap between conventional rigid machines and the compliant, natural motion of biological organisms. By imbuing bioinspired architectures with tunable electrical, thermal, and chemical responsiveness, these emerging technologies are poised to redefine the human–machine interface and enable seamless, adaptive, and intelligent interactions within complex and dynamic environments.

Traditional actuators, ranging from early steam engines to modern miniature motors and gear systems, are based on rigid-body kinematics. Although effective for industrial applications, these actuators are constrained by their high weight, mechanical complexity, and issues such as friction, backlash, and noise. Indeed, when delicate manipulation or adaptation to complex and dynamic environments is required, such systems cannot match the flexibility, redundancy, and high load-to-weight ratio of biological muscles. Artificial muscles overcome these limitations by exploiting the unique properties of soft and active materials to offer key advantages that are unattainable by conventional motors, including intrinsic compliance for safe human–robot interaction, high power-to-weight ratios, silent operation, and distributed actuation. This paradigm shift has transformed actuation designs from employing discrete, rigid joints to continuous, compliant mechanics, enabling the creation of soft, lifelike machines that interact more naturally with their environments.

**Figure 1:**
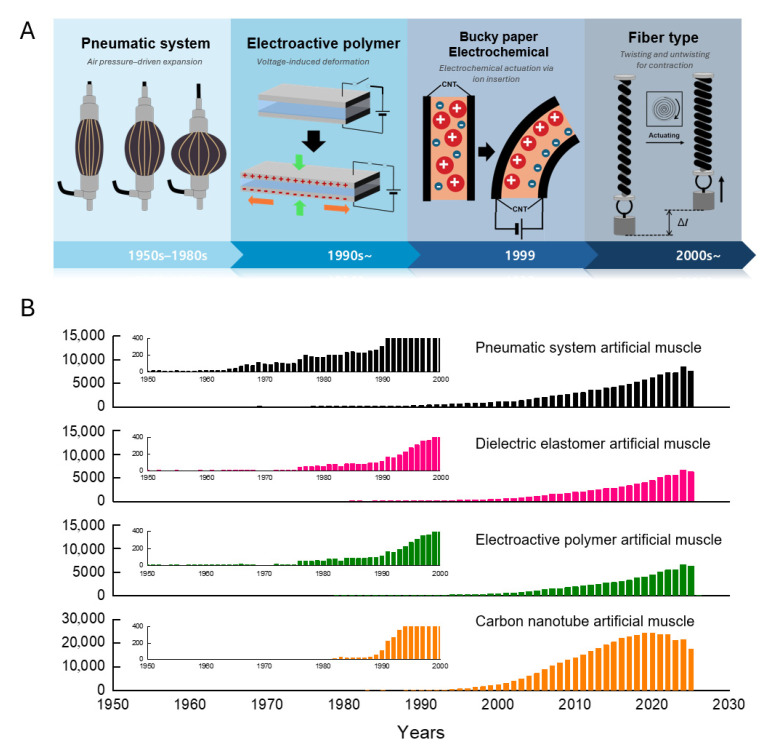
Historical evolution and research trends in artificial muscle technology. (**A**) A timeline illustrating the development of soft actuators from the 1950s to the 2000s, classifying key technologies by their actuation mechanisms: pneumatic systems (pressure-driven), electroactive polymers (voltage-induced), electrochemical bucky papers (ion insertion), and fiber-type actuators (twisting/untwisting). (**B**) Statistical analysis of academic interest showing the annual number of published papers for pneumatic, dielectric elastomer, electroactive polymer, and carbon nanotube artificial muscles.

Early artificial muscles were based on pneumatic systems that used pressurized air to expand elastic bladders, thereby providing simple muscle-like actuation. A major breakthrough was reported in the early 1990s with the development of EAPs, which undergo substantial deformation under applied voltage to closely mimic biological muscle behavior (Figure 1A). Another milestone occurred in 1999, when CNT sheets were demonstrated to act as low-voltage electromechanical actuators, introducing nanomaterials with exceptional strength and conductivity into the field. In the years since, rapid advances have transformed artificial muscles from basic mechanical constructs into intelligent multifunctional systems capable of adaptive and responsive actuation. Figure 1B shows the historical evolution of research subjects and publication trends for four types of biomimetic artificial muscles (pneumatic, DEA EAP, and CNT) from the 1950s to the present, revealing pronounced and accelerating growth in this field. In particular, the sharp increase in the quantity of published research over the past two decades underscores the increasing significance and maturity of artificial muscle technology.

As the development of biomimetic artificial muscles represents a fundamental advance beyond conventional actuation technologies, this review examines the structural and functional principles underlying biomimetic motion and discusses their applications in artificial muscle systems. The remainder of this review is organized as follows: Section 2 explores the diverse actuation mechanisms found in nature that have served as foundational inspiration for engineered systems; Section 3 and Section 4, respectively, focus on bioinspired polymer- and CNT-based artificial muscle systems; Section 5 expands the discussion from material platforms to integrated robotic systems, highlighting the implementation of artificial muscle actuators in emerging soft robotics and bioinspired devices; finally, Section 6 summarizes the conclusions of this review and discusses opportunities for future research.

## 2. Muscle Actuation Mechanisms

### 2.1. Natural Actuation Mechanisms

The artificial muscle actuation is based on the conversion of stored or potential energy into mechanical motion. Nature offers a diverse array of highly efficient actuation strategies that have evolved over millions of years to satisfy the complex demands of movement, manipulation, and survival. Fundamental principles can be extracted from these biological structures and mechanisms, then replicated and adapted to develop synthetic actuation systems.

One of the most representative examples of natural actuation is the human skeletal muscle, which possesses a hierarchical structure extending from bundles of fibers to myofibrils, with contraction initiated at the sarcomere. Neural stimulation induces actin–myosin interactions, which are described by the sliding filament model, to produce contractile forces that are transmitted to bones through tendons to form an efficient lever system, motivating joint movement. By converting chemical energy sources, such as adenosine triphosphate, into precise and controllable mechanical motion, skeletal muscles can serve as a biological basis for the design of artificial muscles.

Muscular hydrostats, such as the octopus arm and elephant trunk, exemplify a fundamentally different actuation strategy [3,4,5]. Without rigid skeletal support, these systems perform elongation, shortening, bending, and torsion solely through the coordinated contraction and relaxation of densely packed muscle fibers oriented longitudinally, transversely, and helically. Contraction of the longitudinal fibers shortens the structure, whereas contraction of the transverse or radial fibers reduces the cross-sectional area and induces elongation under constant-volume constraints. Notably, bending does not result from unilateral longitudinal contraction, which merely causes local bulging, but from the antagonistic interaction between longitudinal fibers on one side and the transverse or radial fibers throughout the organ to maintain its diameter and structural stability. Finally, torsional motion arises from the contraction of peripheral helical fibers, whose large moment arms generate powerful twisting forces. This architecture enables the continuous reconfiguration of shape in which contraction in one direction is balanced by extension in another and offers alternative design principles for artificial muscles, particularly in applications requiring high degrees of freedom, adaptability, and skeletal-independent soft actuation.

Spider silk fibers, which undergo autonomous contraction and torsional deformation in response to variations in ambient humidity to generate substantial mechanical forces, represent another distinctive biological actuation system [6,7,8]. When the relative humidity increases to approximately 70%, spider silk fibers exhibit supercontraction to shrink rapidly in length and release mechanical energy in the absence of external power input. This passive, environment-driven actuation mechanism represents a natural strategy for harvesting environmental stimuli and provides valuable inspiration for designing energy-efficient artificial muscles and soft actuators capable of operating without a direct supply of energy.

Finally, inspiration for artificial muscle designs can also be drawn from plants. For example, the sensitive plant (Mimosa pudica) and Venus flytrap (Dionaea muscipula) exhibit motion driven by near-instantaneous changes in turgor pressure [9,10,11,12]. This mechanism rapidly redistributes water within cells upon external stimulation to generate pressure differentials that induce dynamic movements such as leaf folding or trap closure, representing a form of soft actuation based on fluidic control that offers valuable insights for the development of bioinspired systems.

Taken together, these diverse natural mechanisms demonstrate nature’s vast repertoire of actuation strategies, which encompass the efficiency and precision of skeletal muscle, the versatility of muscular hydrostats, the energy-autonomous behavior of spider silk, and the fluid-driven actuation of plants. Ongoing efforts to replicate or adapt these natural principles in actuator design are driving advances in next-generation artificial muscle technologies and paving the way for efficient and adaptive actuation systems capable of functioning in complex environments.

### 2.2. Artificial Muscle Actuation Mechanisms

Inspired by nature’s example and guided by the principles of physics and materials science, a wide range of fundamental actuation mechanisms have been developed for artificial muscles. Although these mechanisms are driven by diverse stimuli, including heat, electricity, pressure, and chemical reactions, they share a unified physical basis for the conversion of energy into mechanical motion. Artificial muscle technologies can be classified according to the actuation mechanisms they employ, providing an approach for evaluating the associated trade-offs among efficiency, responsiveness, and applicability across diverse technological domains. The principle of stimulus-induced volume change forms the core of artificial muscle technology. Under this principle, an active material expands or contracts in response to an external trigger, such as heat, electrochemical ion insertion, or solvent absorption. This volumetric response must be anisotropic to provide effective actuation, as materials or structures with direction-dependent properties can convert otherwise isotropic expansion into controlled motion. For example, certain polymer fibers exhibit greater radial than axial thermal expansion and may even contract lengthwise when heated, thereby transforming a uniform volumetric change into a useful directional deformation-based actuation mechanism.

**Figure 2:**
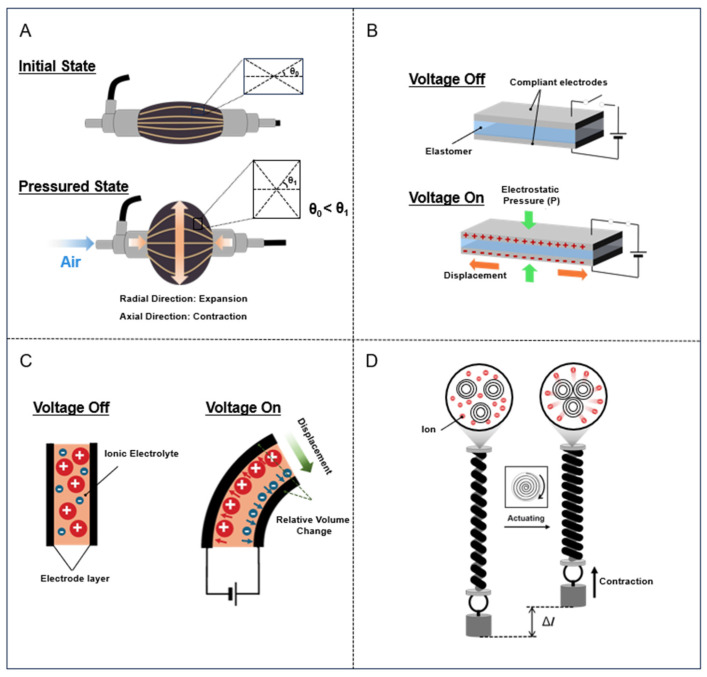
Mechanism of artificial muscles for (**A**) pneumatic, (**B**) dielectric elastomer, (**C**) electro-active polymer, and (**D**) twisted carbon nanotube fiber of yarn.

Despite the diversity of stimuli, the fundamental actuation mechanism across these systems can be unified under a generalized model of ‘stress-induced deformation amplified by structural anisotropy’ [13,14]. In this framework, the actuator generates work (*W*) by expanding or contracting against an external load, but the directionality of this motion is dictated by geometric constraints. The isotropic volume expansion of pressurized air is converted into linear contraction by the inextensible braided mesh, which constrains radial expansion (Figure 2A) [15]. The electrostatic Maxwell stress induces thickness compression, which is converted into planar expansion due to the incompressibility of the elastomer material [16]. Thermal or chemical volumetric changes are converted into tensile or torsional actuation by the helical twist, which acts as a structural bias coupling radial expansion to axial contraction [17]. This unified perspective highlights that performance limits are often defined not just by the active material’s intrinsic energy density but by the efficiency of these structural coupling mechanisms.

Bending motion is typically achieved by incorporating structural asymmetry into the actuator design [13]. For example, a common strategy used to realize bending in fluidic actuators involves reinforcing or fabricating one side of the actuator using a stiffer and less extensible material. When internally pressurized, the more compliant side is elongated to a greater extent than the reinforced side, causing the entire structure to bend away from the latter. A similar mechanism is employed in ionic polymer–metal composites, which apply voltage to drive ion migration toward one electrode, resulting in localized swelling on that side and contraction on the opposite side, thereby realizing an asymmetric dimensional response that produces rapid and reversible bending motion (Figure 2B,C).

Deformations more complex than bending, such as twisting and coiling, can be achieved using torsional actuation mechanisms (Figure 2D) [13,17,18]. Twist-driven actuation, which amplifies small-scale changes into large-scale motions through a helical fiber architecture, is among the most powerful and versatile of these mechanisms. When anisotropic precursor fibers, such as nylon fishing lines or CNT yarns, are highly twisted, the resulting helices store internal strain energy [19]. Upon stimulus-induced volume expansion, such as through heating, this stored energy is released as rapid torsional actuation, producing untwisting rotations capable of driving rotors at exceptionally high speeds. Further coiling of these twisted fibers converts torsional motion into tensile actuation as untwisting draws adjacent coils closer together and contracts the structure along its longitudinal axis. This mechanism can realize stroke strains that exceed 30%, surpassing those of natural muscles. The same principle can also yield tensile expansion by reversing the chirality between the twist and coil. Moreover, guest–host architectures, in which twisted host fibers are combined with diverse responsive guest materials, have extended this concept to realize multifunctional actuation. Collectively, these torsional and tensile mechanisms establish the twist-driven fiber as an adaptable high-performance platform for next-generation artificial muscles.

## 3. Polymer-Based Artificial Muscles

Polymers serve as the cornerstone of many artificial muscle technologies because of their low cost, light weight, high elasticity, intrinsic compliance, and amenability to scalable fabrication and chemical modification. Despite the diversity of polymeric systems, polymer-based actuators can be broadly categorized according to their fundamental actuation mechanisms. The primary distinction can be drawn between pneumatic artificial muscles (PAMs), which employ fluid pressure to generate large contractile forces, electronic EAPs, which are directly activated by electric fields, and ionic EAPs, which operate via ion transport.

### 3.1. Pneumatic Artificial Muscles

**Figure 3:**
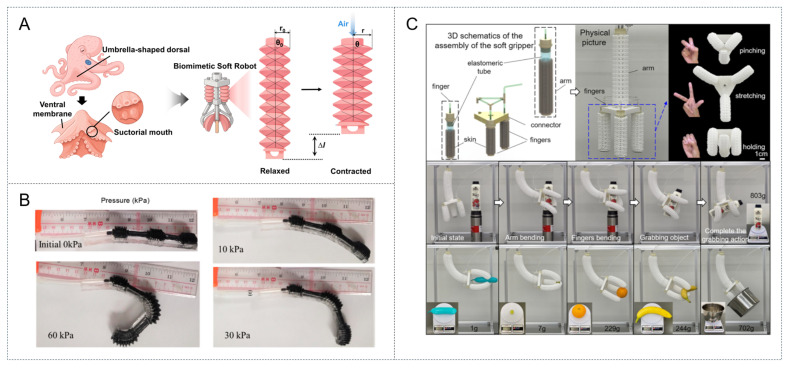
(**A**) Schematic representation of the mechanism for PAM. (**B**) Demonstration of digit mimicking finger action. (Reproduced with permission from Zhao et al. [20]. Copyright 2021, Micromachines; published by MDPI). (**C**) 3D assembly drawings and real-world operating photos of pneumatic tubular grippers for soft robotics applications. (Reproduced with permission from Gao et al. [21]. Copyright 2023, Advanced Intelligent Systems; published by Wiley-VCH GmbH).

Among the most established and widely utilized artificial muscle systems, PAMs are valued for their simplicity, robustness, and muscle-like actuation behavior [20,21]. A typical PAM comprises an elastomeric bladder enclosed within a braided mesh. When the bladder is internally pressurized, its radial expansion is constrained by the mesh, resulting in axial shortening and generating a strong contractile force. This mechanism allows PAMs to exhibit exceptionally high power-to-weight ratios, rapid response times, and outstanding durability, making them well-suited to applications in robotics and assistive devices (Figure 3A). Recent studies have increasingly adopted biomimetic design strategies to enhance the adaptability, dexterity, and multifunctionality of PAMs. These approaches draw inspiration from natural musculoskeletal systems to overcome the inherent limitations of PAMs in terms of stiffness modulation, complex motion control, and environmental adaptability.

Fan et al. created an untethered soft robot that replicated the highly efficient and maneuverable “paddling gait” of the frog by mimicking its dexterous musculoskeletal system to achieve adaptable locomotion [22]. This robot differs from conventional rigid frog robots, which are typically large and structurally complex, as well as existing soft-swimming robots, which typically do not use flexible-leg-based propulsion. The resulting robotic frog was actuated by 12 pneumatic soft actuators (PSAs) that functioned as joints for the hind limbs (hip, knee, and ankle) and forelimbs (shoulder and elbow). These actuators comprised soft silicone rubber with an inextensible layer, such that they either bent or straightened when inflated to replicate limb movements. A key biomimetic feature of this robot was its passive webbed feet made of transparent polyvinyl chloride sheets to mimic the functions of their natural counterparts. The structure of this robot provided numerous advantages: its PSAs were lightweight, rapidly responsive, and inherently watertight; its passive webbed feet automatically opened during the power stroke to maximize thrust and folded during the recovery phase to minimize water resistance; and its soft limbs demonstrated high environmental adaptability, as they were able to deform upon collision and continue functioning. This design resulted in a highly adaptable and agile robot capable of achieving an average speed of 0.1 m s^−1^ and a minimum turning radius of less than one body length (0.15 m).

Zhao et al. created a soft robotic finger that could precisely replicate the complex multi-joint bending motions of the human finger upon which its anatomical structure was based (Figure 3B) [23]. Conventional pneumatic actuators typically bend into a uniform arc, which is “inconsistent with the curved contour of the finger.” Therefore, a biomimetic strategy was applied to design artificial joints that could match the specific anatomical range of motion (ROM) for the metacarpal (MCP), proximal (PIP), and distal (DIP) phalangeal joints of the human finger. The designed soft robotic finger was constructed using three discrete multi-material pneumatic actuators, with each actuator serving as a biomimetic artificial joint. These actuators comprised two components fabricated using materials with different shear moduli: a flexible silicone rubber pneumatic bellows for expansion and a stiffer semi-rigid polydimethylsiloxane (PDMS) frame for restricting that expansion. Each actuator was sized differently for the MCP, PIP, and DIP joints to match the bending capabilities of the human finger. This biomimetic structure was able to accurately mimic natural skeletal bending, allowing the robot to avoid unrealistic uniform bending and achieve a functional ROM (MCP = 36°, PIP = 114°, and DIP = 75°) consistent with the demands of performing everyday activities. Furthermore, this stable and simple multimaterial design realized a soft finger that could successfully grasp various real-world objects, such as beakers and sockets.

Gao et al. designed an actuator based on the muscle–skin biological structure using the principles underlying flexible systems, such as chain mail, that can switch from a soft, flexible state to a rigid, stiff state through structural interlocking or jamming (Figure 3C) [24]. This biomimetic approach was developed to overcome the primary limitations of the fixed stiffness and single predesigned deformation mode associated with conventional pneumatic soft actuators. The result was a highly versatile actuator with programmable deformation and widely tunable stiffness that allows a single robot to adapt to diverse tasks and objects. The design decoupled the actuator into two components: an internal, inflatable elastomeric tube (the “muscle”) and an external three-dimensional (3D)-printed fabric composed of interlocking hollow octahedral unit cells (the “skin”). Bending was realized by fabricating the “skin” using larger unit cells on one side and smaller, more restrictive cells on the other. As a result, the expansion of the internal tube upon inflation was automatically constrained by the “skin,” which forced the actuator to bend toward its more rigid side. This biomimetic separation of “muscle” and “skin” yielded significant advantages: first, the bending stiffness of the resulting actuator was highly tunable within an approximately 56-fold range (from 108 to 5654 N m^−1^) by simply increasing the internal air pressure; second, the deformation was programmable and reusable, allowing the bending direction to be changed by simply rotating the skin around the tube; finally, restitching the skin allowed the actuator mode to be reconfigured from simple bending to S-bending or extension, realizing a single soft gripper that was able to grasp both delicate grapes (as light as 1 g) and large objects (as heavy as 1 kg).

### 3.2. Electronic EAP Artificial Muscles

**Figure 4:**
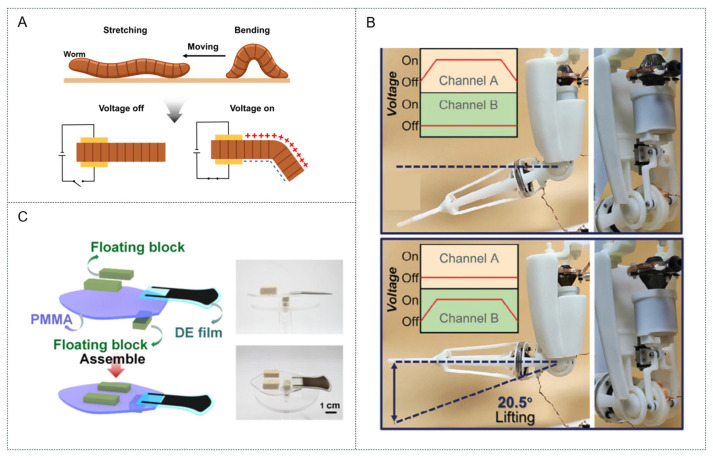
(**A**) Schematic representation of the mechanism for dielectric artificial muscle. (**B**) Action demonstration and its potential applications in human–robot collaboration of a DEA-driven soft mimic robotic arm. (Reproduced with permission from Wang et al. [25]. Copyright 2024, Advanced Functional Materials; published by Wiley-VCH GmbH) (**C**) A fish mimetic robot driven by a GS-DEA tail. (Reproduced with permission from Dong et al. [26]. Copyright 2025, ACS Materials Letters; published by American Chemical Society).

Among the most promising types of electronic EAP actuators, dielectric elastomer technologies and are often regarded as the closest synthetic analogs to natural muscles owing to their exceptional performance. A dielectric elastomer actuator (DEA) is a deformable capacitor consisting of a thin elastomeric film sandwiched between two compliant electrodes (Figure 4A). When a high voltage is applied, the electrostatic attraction between oppositely charged electrodes generates a compressive Maxwell stress that reduces the film thickness while expanding its planar area, thereby producing mechanical actuation. Thus, DEAs have emerged as a highly attractive technology for the development of biomimetic devices, particularly artificial muscles, owing to their profound functional similarities to biological structures and a unique combination of characteristics that closely mirrors that of natural muscles, including inherent softness, lightweight construction, high deformability, and rapid response times [25,26,27]. Furthermore, DEAs exhibit a high energy density and large actuation strains that are similar and in some cases even superior to those of skeletal muscles. Notably, the intrinsic electrical tunability and electromechanical performance of DEAs make them exceptional candidates for replicating the versatile and robust actuation of natural muscular structures. Recent advances have explored novel configurations to realize muscle-like linear contractions; these include “stacked” actuators, which are the closest analog to skeletal muscle and have achieved comparable contraction strains of 24%, as well as “folded” and “helical” designs. Alternatively, expanding DEAs have been configured in antagonistic pairs to directly mimic the biceps–triceps relationship and thereby power robotic limbs and prosthetics [25,26,27].

Recent advances in DEAs have focused on mitigating hysteresis losses, dielectric breakdown, and output limitations to enable their use in biomimetic soft robotics and human–robot collaboration. Traditional acrylic tape-based DEAs exhibit high permittivity but suffer from viscoelastic degradation, which limits their operational stability. Wang et al. addressed these challenges by developing a dual-cone dielectric elastomer actuator (DCDEA) composed of acrylic block copolymer films incorporating poly(n-butyl acrylate) soft segments for high strain resistance and polymethyl methacrylate hard segments for enhanced force output and reduced hysteresis (Figure 4B) [28]. The DCDEA was applied in a soft robotic arm mimicking the biomechanics of the human arm to ensure compliant and safe interactions in human–machine collaboration environments; it incorporated a central spacer to store elastic energy and realize significant bidirectional strokes with a load-to-weight ratio of 1300. When combined with crank–slider and nut–screw mechanisms, the DCDEA was able to drive a soft robotic arm through human-like lifting and twisting motions, in one case transporting a 10 g load with an energy consumption of only 35 mJ and achieving an energy density of 42.1 J kg^−1^. Furthermore, the DCDEA-based arm was enclosed within a flexible replica-cast shell to ensure safety during combined human–robot operations, such as precision assembly. The selected acrylic block copolymer actuator material demonstrated exceptional performance, achieving an actuated strain of 304% and blocked stress of 168 kPa when prestretched, thereby realizing a high-performance soft-driven actuator unit.

However, interfacial stress often causes asymmetric-structured DEAs to fail when attempting out-of-plane actuation, which is essential for grasping and locomotion. Dong et al. accordingly designed a series of gradient-structured DEAs inspired by the three-order-of-magnitude difference between the elastic modulus of bone and cartilage (Figure 4C) [29]. These actuators comprised tunable polyhexyl diacrylate elastomers (PHDEs) with moduli adjusted from 0.17 to 107 MPa by varying the applied cross-linking ratio of CN9021 to propoxylated neopentyl glycol diacrylate as well as the monomer composition. The gradient structure was realized by inserting multiple buffer layers, each with a stepwise increase in modulus, between the soft actuator and its stiff substrate, mimicking the bone–cartilage interface. This biomimetic gradient design effectively reduced the interfacial shear stresses and considerably improved actuator durability. A buffer layer of stiffening films—ranging from PHDE-9 (active region, modulus of 1.1 MPa) to PHDE-28 and 43 (modulus of 13.7 and 107 MPa, respectively) laminated on polyethylene terephthalate via dry stacking—enabled a curvature of 1.66 cm^−1^, output force of 30.8 mN at 70 V μm^−1^, and response times of 100/66 ms while maintaining performance over >100,000 cycles. This excellent performance was attributed to the reduction in interfacial shear stresses to below the bond strength limits, retaining 85% of curvature even under 10 Hz operation. When applied as a biomimetic robotic fish fin, the gradient-structured DEA demonstrated long-term swimming capabilities with enhanced durability and stable cyclic performance.

### 3.3. Ionic EAP Artificial Muscles

**Figure 5:**
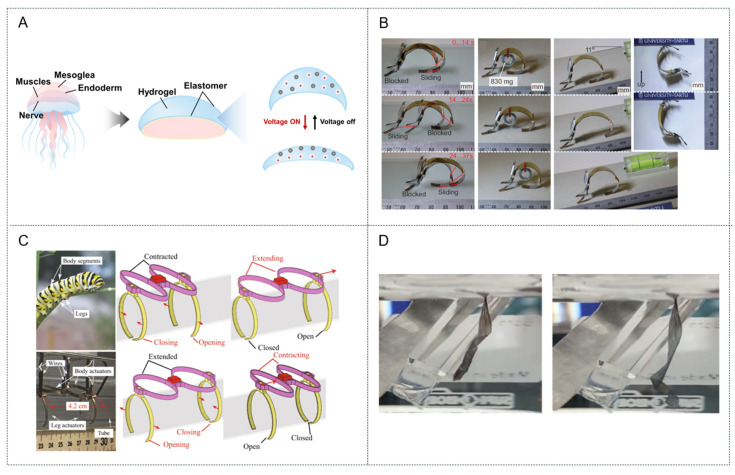
(**A**) Schematic representation of mechanism for ionic EAP artificial muscle. (**B**) Movement of IEAP robots imitating inchworm. (Reproduced with permission from Must et al. [29]. Copyright 2015, Advanced Engineering Materials; published by Wiley-VCH GmbH) (**C**) 3D-printed IPMC soft crawling robot inspired by a caterpillar. (Reproduced with permission from Carrico et al. [33]. Copyright 2019, Scientific Reports; published by Springer Nature Limited) (**D**) PPy micro ribbon artificial muscle, which mimics the morphology of natural muscle fibers. (Reproduced with permission from Berogoi et al. [32]. Copyright 2022, Scientific reports; published by Springer Nature Limited).

In contrast to electronic EAP actuators, such as DEAs, ionic EAPs operate via electrically induced ion migration within the polymer matrix, enabling actuation at low and safe voltages (Figure 5A) [30,31]. Among ionic EAPs, ionic polymer–conductive material composite systems, which typically comprise a polyelectrolyte membrane coated with flexible conductive electrodes, are the most extensively studied. When a low voltage (<7 V) is applied to this composite, solvated cations migrate toward the cathode, where they induce localized swelling on one side and contraction on the other, thereby bending the entire strip. This mechanism has been exploited in biomimetic systems, such as beetle-inspired flapping micro-air vehicles, in which lightweight low-voltage actuation is essential.

In this context, Must et al. developed a supercapacitor-like ionic EAP laminate incorporating activated carbon electrodes, an ionic liquid electrolyte, and compliant gold current collectors to power an 830 mg inchworm-inspired crawler that represented the lightest power-autonomous EAP robot reported at the time (Figure 5B) [32]. Mimicry of inchworm locomotion using a simple bending–relaxation gait achieved effective rectilinear (straight-line) motion on a dry surface. The key structural advantage of this design was the high bending modulus (stiffness) of the single ionic EAP actuator, which allowed it to serve as both the “muscle” and the primary structural “body” of the robot, supporting its own weight as well as a battery and even a payload. Furthermore, the actuator was intentionally preshaped into a nonplanar arc to prevent undesirable buckling and ensure bending in a controlled and predictable direction. Operating at only a few volts through pulse–width modulation control, the non-planar actuator produced a looping undulatory motion on smooth surfaces in open air to achieve a top speed of 2.2 m h^−1^ while carrying an 890 mg payload. Notably, this design bypassed the dehydration requirements of traditional devices for stable in-air operation and demonstrated a high capacitance in excess of 150 mF cm^2^. Similarly, Carrico et al. introduced a biomimetic robot inspired by caterpillar-like organisms using ionic polymer–metal composites at low voltages (<3 V) to implement the unique and effective “inching” locomotion method (Figure 5C) [33]. This robot was designed to “grip” onto a cylindrical tube and “inch” its way along its surface using 3D-printed linear actuators as “body segments” and modular grippers as “legs.” A major advantage of this caterpillar-inspired structure is its ability to perform complex sequential gaits in which its body segment actuators contract and extend while its front and rear legs alternately grip the surface. Another key advantage of this biomimetic design is its modularity, which allows components to be assembled into various configurations to create reconfigurable robots of varying lengths.

Additionally, Yeom et al. fabricated a biomimetic jellyfish robot using ionic polymer–metal composite EAP actuators that were thermally treated at 80 °C for one hour to form them into permanent hemispheres [34]. The resulting robot comprised eight curved legs actuated by ionic polymer–metal composite EAPs and interconnected by a cellophane skirt to generate a jet-like thrust through bell pulsation, mimicking the propulsive mechanism employed by jellyfish. A bioinspired electrical driving signal characterized by a fast-pulse–slow-recovery phase ratio of 3:7 was used to actuate the robot and proved to be considerably more effective than conventional sinusoidal inputs. Using this actuation mechanism, the robot achieved a peak upward velocity of 0.057 mm s^−1^ at 1.0 Hz and a thrust force of 0.87 gf per cycle, exhibiting optimal performance near the 2.2 Hz natural resonance frequency with sustained locomotion in water.

Finally, Berogoi et al. developed a bioinspired artificial muscle that functionally mimicked natural muscle by integrating actuation with intrinsic mechanical sensing (Figure 5D) [35]. This approach replicated the fibrillary morphology of natural muscle fibers using electrospun microstructures to emulate the dual functionality of mammalian muscles, which act as both actuators and sensory receptors. The primary advantage of this fibrillary design is its large active surface area, which was shown to facilitate rapid ionic exchange and highly responsive actuation. The key functional advantage of the overall approach is its ability to simultaneously generate movement and “sense the effort” of a load, similar to proprioception. As a result, this device demonstrated muscle-like behavior, in which the actuation amplitude decreased as the applied load increased, while precisely quantifying the load with a high sensitivity of up to 7.2 mV mg^−1^.

## 4. CNT-Based Artificial Muscles

**Figure 6:**
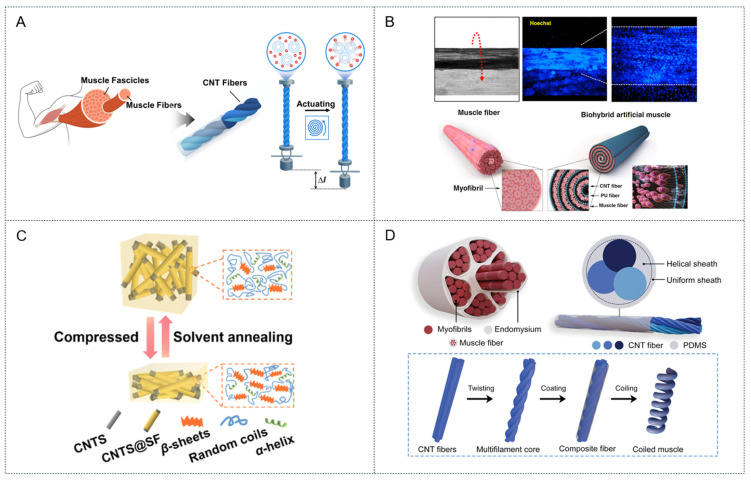
(**A**) Schematic representation of mechanism for CNT fiber or yarn types artificial muscle. (**B**) Schematic illustration of biohybrid artificial muscle, and 3D fiber structure fabrication using a biscrolling C2C12 cell-laden PU/CNT matrix. Blue fluorescence indicates each cell nucleus stained with Hoechst dye. (Reproduced with permission from Jang et al. [34]. Copyright 2021, Microsystem & Nanoengineering; published by Springer Nature Limited) (**C**) Schematic image for hybrid sponge composed of silk fibroin confined within a porous CNT network. (Reproduced with permission from Yang et al. [36]. Copyright 2025, ACS Nano; published by ACS) (**D**) Schematic of actuation process of natural muscle, and myofibril-inspired CNT yarn artificial muscle. (Reproduced with permission from Zhu et al. [37]. Copyright 2025 Small; published by Wiley-VCH GmbH).

Although polymer-based systems provide a versatile foundation for artificial muscle technologies, CNT-based actuators represent the cutting edge of performance by often surpassing both natural muscles and other synthetic actuators in key metrics, such as specific work and power density. The unique combination of exceptional mechanical strength, high electrical and thermal conductivities, and large specific surface area makes CNTs ideal materials for next-generation high-performance actuators. Previous studies have shown that CNT-based actuators can be fabricated in two basic configurations: composites and twisted yarns. Composite systems are created by embedding dispersed CNTs in various polymeric or functional matrices to form structural architectures tailored for specific applications. Yarn systems are created by twisting CNTs into helical architectures that are not merely structural but also serve as the core mechanism for amplifying nanoscale dimensional changes into powerful macroscopic actuation, thereby achieving higher actuation performance than composite systems (Figure 6A).

### 4.1. Composite CNT Artificial Muscles

Composite CNT actuators capitalize upon the exceptional intrinsic properties of CNTs by integrating them into polymers or other functional matrices. This strategy addresses key challenges, such as processability, scalability, and environmental stability, while enabling multifunctional capabilities that pure CNT yarns alone cannot readily achieve. One promising approach embedded a composite fiber comprising an aligned network of graphene oxide and multi-walled CNTs within a polyurethane matrix to mimic the rigid β-sheet and amorphous domains of natural spider silk [7]. This bioinspired structural design yielded an exceptional gravimetric toughness of 494 J g^−1^ that far exceeds that of natural spider silk (165 J·g^−1^). Functionally, this composite fiber reproduced the characteristic supercontraction of spider silk, exhibiting up to 60% contraction in response to humidity and thereby demonstrating a powerful moisture-driven actuation mechanism. The material also exhibited remarkable elasticity, sustaining approximately 80% of its fully reversible strain without mechanical degradation. Similarly, Weng et al. demonstrated a multifunctional composite actuator inspired by the network structure of cobwebs to address the poor mechanical properties typical of pure CNT films [38]. This approach utilized bacterial cellulose (BC) as a binder to create a mechanically robust 3D CNT-BC network that was further enhanced by the in situ polymerization of polyaniline (PANI). The resulting PANI@CNT-BC film was used as the core of a bilayer actuator exhibiting a multi-responsive actuation mechanism. The hydrophilic PANI@CNT-BC layer functioned as the active layer for humidity-driven actuation by swelling through hygroexpansion, whereas the polyethylene terephthalate/poly(3,4-ethylenedioxythiophene) polystyrene sulfonate substrate, which had a high coefficient of thermal expansion, functioned as the active layer for light and electrical stimuli-driven actuation by asymmetric thermal expansion. This device demonstrated excellent performance with bending curvatures up to 1.22 cm^−1^ (electrically driven) while integrating advantageous secondary functions, such as electromagnetic interference shielding (25.8 dB) and energy storage (235.7 mF cm^−2^).

Furthermore, inspired by the hierarchical architecture of natural skeletal muscle, Jang et al. developed a biohybrid artificial muscle by integrating living cells into a synthetic nanofibrous scaffold composed of hydrophilic polyurethane and CNT nanofibers to mimic the native actin–myosin myofilament arrangement supporting muscle cells (Figure 6B) [39]. Skeletal myoblasts seeded onto this matrix were assembled into a 3D fiber bundle using a biscrolling technique, thereby replicating the bundled structure of muscle tissue. The living cells differentiated into aligned multinucleated myotubes within this biomimetic scaffold; upon electrical stimulation, they generated reversible contraction of the entire biohybrid fiber, resulting in a functional, cell-actuated artificial muscle. Another actuation paradigm was inspired by the remarkable load-bearing capacity of insects, such as ants (Figure 6C) [36]. Yang et al. employed a hybrid sponge composed of silk fibroin (SF) confined within a porous CNT network to harness the pressure-induced conformational transition of silk fibroin and thereby transform the SF proteins from flexible α-helices/random coils into rigid β-sheet structures under a mild compressive stress (<10 MPa). This transition effectively “freezes” the sponge into a temporarily ultrastiff (>10 MPa) state, enabling the lightweight composite to support loads greater than 10,000 times its own weight.

### 4.2. CNT Fiber- and Yarn-Based Artificial Muscles

Natural muscles rely on hierarchical organizations and helical filament arrangements to amplify molecular interactions into macroscopic contraction. Similarly, CNT yarn actuators employ twist- and coil-induced architectures to convert minute dimensional changes in CNTs into significant torsional and tensile strokes that can surpass the performance of biological muscles [17,18,40]. One strategy for doing so at the macroscopic level replicates the architecture of natural muscle by bundling multiple torque-stabilized CNT filaments to form artificial muscle fascicles [41]. This hierarchical design, which is analogous to the natural assembly of myofibrils into fascicles, substantially amplified the generated force and work capacity. For example, a 16-ply artificial muscle fascicle was able to generate an isometric force exceeding 538.4 mN, more than six times that of a single filament, while maintaining a large contractile strain in excess of 30%. Moreover, these fascicles exhibited an integrated energy storage capability, allowing them to function as supercapacitors with significantly greater capacities than individual CNT filaments. Another strategy at the single-fiber level draws inspiration from the endomysium (Figure 6D) [37], which is the connective tissue layer surrounding individual myofibrils, by encapsulating a multifilament CNT core (analogous to the myofibril) in a polymer (e.g., PDMS) sheath (analogous to the endomysium) that functions not as a passive coating, but as an active component to enhance torque generation and actuation performance. This design produced an electrothermally driven artificial muscle capable of delivering a contractile stress of 40.1 MPa and work capacity of 1.45 J g^−1^ under heavy loads.

Beyond structural mimicry, recent studies have advanced toward functional biomimicry by emulating the energy conversion processes of natural muscles [42]. This approach has produced systems integrating energy generation, storage, and actuation within a single trifunctional CNT yarn, effectively replicating the biological mechanism by which muscles convert chemical energy into electrochemical ion gradients to drive contraction. Such systems have self-generated up to 360 mV and utilized this internally generated energy to induce a tensile stroke of 0.14% within 2.3 s, thereby mimicking the rapid and autonomous response of natural muscle.

Despite these remarkable actuation capabilities, CNT yarn artificial muscles face significant challenges regarding long-term reliability and scalable fabrication. A primary failure mechanism is the mechanical hysteresis and creep deformation observed under high-load cycling, which originates from the irreversible slippage of CNT bundles within the yarn structure [43,44]. Unlike covalently cross-linked polymers, the van der Waals forces holding the CNT bundles together can allow for gradual structural relaxation, leading to a loss of actuation stroke over time (fatigue). Furthermore, the “twist instability”—where highly twisted yarns tend to snarl or partially untwist—poses a reliability issue for continuous operation. From a manufacturing perspective, while lab-scale spinning is well-established, the large-scale fabrication of kilometer-long CNT yarns with uniform twist density and consistent electrochemical properties remains a critical bottleneck impeding industrial adoption [45].

To contextualize the unique position of CNT actuators, it is essential to compare their performance metrics with those of established polymer and fluid-driven systems. Traditional PAMs are widely utilized for their high force output and safety; however, they require bulky external compressors and valves, which severely limit their system-level energy density and portability. Similarly, DEAs and other EAPs offer large actuation strokes (>20%) and rapid response times that closely mimic natural muscle. Yet, DEAs typically necessitate high driving voltages (>1 kV), posing safety risks and requiring complex high-voltage amplifiers, while ionic EAPs are often constrained by low blocking stresses and slow electromechanical response speeds due to ion diffusion limits.

CNT yarn actuators bridge these gaps by delivering superior specific stress generation combined with high thermal and electrical conductivity that enables faster frequency responses. Although CNT systems currently face challenges regarding material cost and fabrication complexity compared to scalable polymer fibers, their ability to generate high work densities at low voltages (for electrochemical types) or high frequencies (for electrothermal types) makes them indispensable for applications requiring compact, high-power actuation where bulky pneumatics or high-voltage electronics are impractical.

## 5. Artificial Muscles for Robotics

The ultimate application of artificial muscle technologies is their integration into functional robotic systems capable of meaningful and autonomous operations. The distinctive properties of these actuators, such as compliance, low weight, high power density, and silent operation, have led to the emergence of a new generation of robotic systems exhibiting enhanced functionality and adaptability. From a practical standpoint, however, replacing traditional electromagnetic motors requires a critical evaluation of system-level energy density and actuation cost. While PAMs offer high force-to-weight ratios at the component level, they suffer from low system-level energy density due to the necessity of heavy external compressors and valves, which restricts their use in untethered soft robotics [46]. Similarly, DEAs exhibit high theoretical energy densities (>500 J kg^−1^) comparable to electromagnetic motors; however, their practical implementation is often hindered by the complexity and weight of the high-voltage amplifiers required for operation [47]. In this context, CNT and polymer fiber actuators present a unique advantage by offering high specific work densities without the need for bulky fluids or complex high-voltage electronics, although they face challenges regarding thermal efficiency and material costs [13]. Recent innovations have also begun to integrate energy-harvesting functionalities directly into these fiber-based actuators, enabling self-powered systems that address the power autonomy challenge [42]. Despite these system-level challenges, artificial muscles remain an essential technology for Soft Robotics, an emerging and rapidly advancing field that aims to construct robots from highly compliant and deformable materials. Drawing direct inspiration from soft-bodied organs in nature, such as the octopus tentacle and elephant trunk, soft robotics relies on these actuators to manipulate flexible structures. Critically, the inherent compliance of artificial muscles enables soft robots to exhibit dexterous motion, adapt to unstructured environments, and safely interact with humans, which are essential prerequisites for robots in collaborative and medical applications.

### 5.1. Soft Robotics

Octopus tentacles have long served as a major source of inspiration for soft robotic manipulators and grippers owing to their muscular structure, which lacks a skeleton and provides unique motor skills, including the ability to bend in all directions, produce rapid elongations, and actively vary stiffness. This functionality can be achieved using densely packed muscles in orthogonal (transverse and longitudinal) arrangements that act antagonistically while maintaining a constant volume, which represents the primary advantage of this biomimetic design. Modeling has demonstrated that this orthogonal mechanism allows small contractions of the transverse muscles to produce large, passive elongations along the longitudinal axis. Therefore, robots incorporating octopus-inspired actuators can achieve omnidirectional bending and stiffness control by arranging artificial muscles in similar antagonistic, hydrostatic structures [48,49,50]. For example, Wu et al. developed a deformable silicone suction disk equipped with an array of suctorial mouths actuated by a 3D-printed linkage mechanism [49]. This configuration enabled powerful multimodal grasping by combining strong suction (~100 N) with adaptive wrapping to effectively grasp a wide variety of objects (flat, irregular, or scattered) and even handle moving organisms such as turtles on both smooth and rough surfaces. A critical innovation associated with this study was its use of a tactile sensing mechanism in which contact with an object blocks certain suction cups, thereby altering the overall fluid flow rate measured by a turbine flowmeter to provide tactile feedback.

Similarly, Shi et al. presented a soft somatosensory actuator inspired by the multifunctional architecture of octopus tentacles that mimics their integration of neuromuscular system (actuation), nerve cords and receptors (sensing), and biological tissue systems (self-healing) [50]. An artificial architecture was developed to provide three components chemically: a photo-responsive anthracene in a PDMS matrix to mimic the neuromuscular effectors, thereby enabling rapid, light-driven bending actuation (10° s^−1^); high-conductivity sliding silver nanowires embedded on the surface to mimic nerve cords that slide to change resistance and provide distinctive intrinsic strain sensitivity (GF 90.88) as the actuator bends; and finally, a matrix containing multiple dynamic interactions (e.g., disulfide bonds, hydrogen-bonding) that mimic biological tissue systems. Notably, this integrated design produced a monolithic material that can autonomously heal damage (with 92.2% efficiency) while actuating and sensing its own motion.

Finally, Lee et al. proposed a soft adhesion actuator that emulated the unique ability of the octopus sucker to achieve robust, switchable adhesion in both dry and aquatic environments [51]. This actuator mimicked sucker morphology using a soft, inflatable dome that was expanded by pneumatic actuation to produce a significant pressure differential relative to the ambient environment and thereby achieve a powerful suction force. This mechanism demonstrated exceptional performance by achieving high suction forces of approximately 26 N (dry) and 45 N (underwater). Furthermore, this artificial muscle integrated biomimetic nerve-like functions via 3D spray-coated CNT strain sensors that enabled the actuator to perceive the object weight and center of gravity through machine learning, demonstrating a sophisticated, integrated system for grasping and manipulation.

Insects are another major source of inspiration for small, fast, and agile soft robots because of their exceptional agility, efficiency, and ability to navigate constrained environments [52]. Inspired by the rapid muscle contractions and gait patterns of insects such as caterpillars, Zhu et al. developed an untethered insect-scale soft robot measuring only 15 mm in length and weighing only 450 mg [53]. The remarkable performance of this robot, including an ultrafast speed of approximately 4.0 body lengths per second, was attributed to its use of a 3D-printed dielectric actuator that functioned as an artificial muscle. This actuator operated at frequencies up to 760 Hz and exhibited remarkable durability by maintaining stable performance over one million actuation cycles. The locomotion of this robot was achieved using an asymmetric barb-like leg structure that efficiently converted the high-frequency oscillations of the actuator into forward motion via directional friction.

Furthermore, Tsai et al. demonstrated a miniature insect-scale jumping robot that leveraged locust-inspired kinematics and coiled artificial muscle actuators produced by additive manufacturing to overcome the mass inefficiency and design constraints associated with conventional manually assembled jumping robots [54]. The design of this robot mimicked the power amplification of the locust using a monolithic, fully elastomeric body as a compliant four-bar linkage to store high elastic energy throughout its structure. This stored energy was released using a latch-triggering mechanism actuated by a lightweight, nylon-coiled artificial muscle that provided a higher work capacity and more compact solution for initiating impulsive energy release than a bulky motor. This combination of bioinspired elastomeric energy storage and coiled artificial muscle triggering resulted in a 0.216 g robot capable of jumping 60 times its body size.

### 5.2. Advanced Biomimicry: Replicating Locomotion and Manipulation

The application of advanced biomimicry in artificial muscle research attempts to replicate not only the morphology but also the complex functional mechanisms of living organisms to enhance robot performance. The development of novel actuators that emulate biological structures and dynamics has allowed researchers to create soft, resilient, and autonomous robots capable of swimming, flying, and grasping in previously unattainable ways.

Aquatic environments present unique challenges for robotic locomotion, requiring efficiency, quiet operation, and adaptability. Li et al. accordingly developed a jellyfish-like artificial muscle actuator to power an untethered underwater robot [55]. This actuator consisted of a dielectric elastomer–hydrogel composite with a high water content (83.3%) that formed an ultrasoft, water-rich structure. The hydrogel served as both a soft skeletal support and a transparent electrode, and the dielectric elastomer membrane generated voltage-driven deformation, enabling controllable forward and backward propulsion. This untethered robot achieved a maximum speed of 0.91 cm s^−1^ and an operational endurance of 15.7 min when powered by a 500 mAh lithium-ion battery. Similarly, Whener et al. developed an octopus-inspired soft robot comprising a fully soft body actuated by the catalytic decomposition of an onboard monopropellant and microfluidic logic circuits to regulate the pneumatic inflation patterns for coordinated motion [56]. Furthermore, Koh et al. developed an insect-scale jumping robot by elucidating the biomechanics of the water strider [57]. Their analysis revealed that water striders maximize momentum transfer for jumping on water by rotating their legs to apply a gradually increasing force that exploits, but does not break, the water surface tension limit. This surface-tension-dominant propulsion was mimicked in their 68 mg robot by employing a flea-inspired impulsive mechanism rather than direct actuation; it consisted of a torque reversal catapult mechanism triggered by an artificial muscle made from a coiled shape actuator. This muscle–catapult system was shown to be advantageous because it generated a gradually increasing torque profile that matched the ability of the water strider to push the water surface tension to its limit without rupture, thereby enabling the robot to jump on the surface of water.

On land, the exceptional mobility of the gecko, which is characterized by the coordination between cyclical lateral swinging of the trunk and limb movements, inspired the development of a gecko-like robot equipped with a flexible spine actuated by shape memory alloy springs [58]. This approach replicated the sprawling posture, single-peak C-shaped spine curvature, and trotting gait of the gecko using a kinematic model to describe the relationship between the leg joint variables and spinal deflection angle. The experimental results indicated that the flexible-spine configuration enabled a longer stride length, higher locomotion speed, and smaller turning radius than a rigid spine, thereby enhancing the agility and maneuverability of the robot. Furthermore, Liu et al. were inspired by the coordinated interaction between the actuating muscles and adhesive toe structures of the gecko to develop a smart adhesive film for the adaptive manipulation of objects with unknown surface morphologies [59]. This system integrated a magnetic artificial muscle with mushroom-shaped microstructures that provided controlled adhesion. When subjected to a magnetic field, the film conformally attaches to target surfaces with flat or curved geometries and smooth or rough textures, enabling the robot to grip, transfer, and release unfamiliar objects without relying upon complex image recognition or sensing systems. Notably, the gripped object remained attached even when the magnetic field was abruptly removed owing to the inherently adhesive properties of the film microstructure. This approach broadens the applicability of gecko-inspired adhesion from specific surfaces to a wide range of unknown morphologies, paving the way for versatile and reliable robotic gripping systems.

Finally, insects exhibit exceptional flight capabilities during aerial locomotion, including rapid maneuvers and efficient collision recovery. Inspired by the hindwing of the scarab beetle, Hou et al. developed a lightweight and intelligent membranous wing integrating aerodynamic load bearing, piezomechanoreceptive sensing, and self-powered functionalities [60]. This bionic wing reproduced the semi-tubular costa structure of the beetle by incorporating three distinct membranous regions (anal, medial, and apical) fabricated through heat lamination of multilayer composite materials. The resulting wing exhibited an aerodynamic performance that closely mirrored that of a real beetle. Functioning as a piezomechanoreceptive sensor, this wing was able to detect flapping frequency, wing deformation, and collision events through the voltage signals generated by embedded piezoelectric materials. Notably, further investigations of rhinoceros beetles have revealed that their origami-like wing folds play a critical shock-absorbing role during in-flight collisions [61]. Upon impact, such wings collapse along these predefined folds and subsequently spring back within a single stroke, dissipating energy and allowing rapid flight recovery. Therefore, this biological mechanism has been replicated in beetle-inspired flapping-wing robots to ensure stable flight even after collisions.

## 6. Conclusions

**Figure 7:**
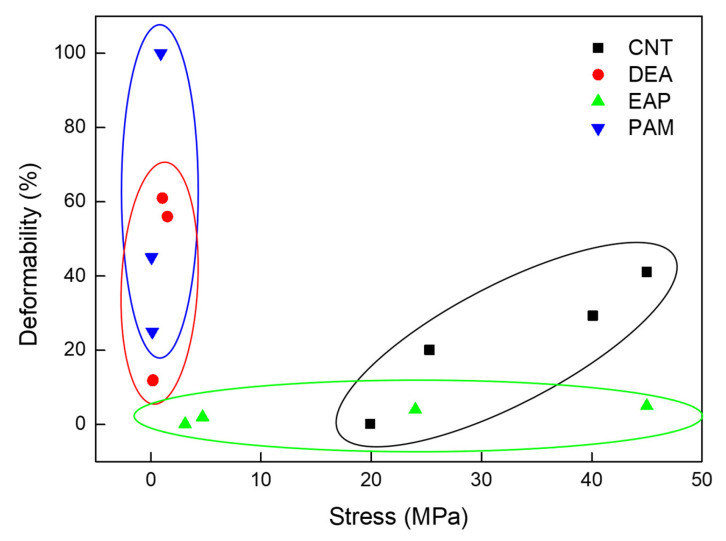
Performance comparison of various artificial muscle technologies represented actuation stress versus actuation deformability. The plot categorizes the artificial muscles into three distinct performance regimes for CNT [37,41,42,63], DEA [64,65,66,67], EAP [68,69], and PAM [70,71].

Advances in biomimetic artificial muscles represent critical technological breakthroughs and reflect a deep understanding of how nature achieves movement, adaptability, and intelligence through hierarchical structural and functional principles. Rather than merely replicating the biological form, the biomimetic approach seeks to capture the functional features of living actuation systems in which materials, structures, and controls operate as an integrated whole. As discussed in this review, polymer-based artificial muscle systems, such as PAMs, dielectric elastomers, and ionic EAPs, embody nature’s strategy of combining softness and responsiveness to achieve efficient and reversible deformation. Furthermore, CNT-based systems have demonstrated how hierarchical architectures reminiscent of muscle fiber organization can amplify nanoscale interactions into macroscopic power outputs to achieve performance levels comparable to or exceeding those of natural tissues (Table 1). To visualize the distinct performance regimes of the discussed technologies, we present a performance map correlating actuation stress and strain in Figure 7.

Challenges to the practical deployment of these technologies are their long-term reliability and failure management. Polymer-based systems, particularly DEAs, are prone to dielectric breakdown under high-voltage operation and viscoelastic creep over time [47]. Similarly, CNT-based actuators often suffer from performance degradation due to moisture absorption or structural fatigue at high actuation frequencies, where irreversible untwisting or bundle slippage can occur [72,73]. Future development must therefore prioritize not only the maximization of work density but also the characterization of failure modes to ensure operational stability in real-world environments.

Jellyfish-like swimmers, octopus-inspired grippers, gecko-mimetic manipulators, and beetle-inspired aerial robots exemplify the manner in which artificial muscles can reproduce the synergy of structure, function, and control that defines biological locomotion. However, several challenges must be overcome before artificial muscles can fully emulate the autonomy and resilience of their biological counterparts. Future biomimetic research should focus on reproducing the self-adaptive intelligence of natural muscles using integrated sensing, self-repair, and energy management capabilities. In particular, the development of closed-loop learning-based control architectures inspired by neural feedback is essential for realizing true biohybrid functionality. Ultimately, biomimetic artificial muscles offer a unified framework for understanding and recreating the mechanics of living creatures. By continuing to explore natural principles of hierarchical organization, multifunctionality, and energy efficiency, progress is being made toward the development of autonomous, lifelike machines that reflect the fundamental dynamics of natural mechanical systems.

## Figures and Tables

**Table 1:** Summary of biomimetic artificial muscles.

Biomimetic	Actuation Mechanism	Movement	Deformability	Work	Ref
Frog	Pneumatic	Bending	160°	40 N mm	[22]
Joints	Pneumatic	Bending	114°	-	[23]
Muscle	Pneumatic	Bending	180°	6.81 N	[24]
Octopus	Pneumatic	Tensile	41.1 mm	46.7 N	[49]
Muscle	DEA	Tensile	304%	42.1 J kg^−1^	[28]
Bone and cartilage	DEA	Bending	1.66 cm^−1^	30.8 mN	[29]
Muscle	DEA	Tensile	~65%	2.5 N	[26]
Inchworm	IEAP	Bending	45 m^−1^	20 mN	[32]
Jellyfish	IPMC	Bending	~30 mm	-	[34]
Manta ray	IEAP	Bending	12.5 mm	0.831 mN	[62]
Spider silk	Swelling (CNT composite)	Tensile	60%	610 kJ m^−3^	[7]
Cobweb	Humidity Temperature Electrical (CNT composite)	Bending	−0.66 cm^−1^ 0.56 cm^−1^ 0.53 cm^−1^	241.56 μW cm^−2^	[38]
Muscle	Cell contraction (CNT composite)	Tensile	2.73 ± 0.27 μm	-	[39]
Silk	Recovery (CNT composite)	Tensile	90%	-	[36]
Muscle	Electrochemical (CNT composite)	Tensile	>30%	>0.5 N	[41]
Muscle	Electrochemical (CNT composite)	Tensile	29.3%	1.45 J g^−1^	[37]

## Data Availability

No new data were created or analyzed in this study.
